# A relapsed *Pseudomonas stutzeri* prosthetic valve endocarditis: a case report and review of the literature

**DOI:** 10.1186/s13256-021-03084-x

**Published:** 2021-10-10

**Authors:** Mayyadah Alabdely, Mohammed Alazmah, Bandar Alamro, Mohamad S. Alabdaljabar, Magid Halim

**Affiliations:** 1 Department of Medicine, King Faisal Specialist Hospital and Research Center, Riyadh, Saudi Arabia; 2 Heart Center, King Faisal Specialist Hospital and Research Center, Riyadh, Saudi Arabia

**Keywords:** *Pseudomonas stutzeri*, Infective endocarditis, Prosthetic valve, Diagnosis, Infection, Case report, Saudi Arabia

## Abstract

**Background:**

*Pseudomonas stutzeri* is a nonfluorescent denitrifying bacterium widely distributed in the environment, and it has also been isolated as an opportunistic pathogen from humans. It is a Gram-negative bacterium and a common inhabitant of soil and water.

**Case presentation:**

We report the case of a 51-year-old arab gentleman who has systemic lupus erythematous complicated by lupus nephritis and underwent renal transplantation twice. He underwent mitral valve replacement and 4 years later was diagnosed with prosthetic valve endocarditis caused by *Pseudomonas stutzeri*.

**Conclusions:**

Literature review was conducted and revealed that this pathogen may be of a particular medical relevance in immunocompromised patients. Our case proves that early infection and relapse despite optimal antibiotics course are possible outcomes of *Pseudomonas stutzeri* endocarditis. To the best of our knowledge, this is the second case of fulminant early prosthetic valve endocarditis occurring only 1 month post-cardiac surgery with relapse despite a complete antibiotics course.

## Introduction

Infective endocarditis (IE) is a rare yet serious condition that is associated with poor prognosis if not appropriately managed. The most common causative organisms are Gram-positive bacteria; however, Gram-negative counterparts can also lead to IE, and are associated with poor prognosis [1]. In the Gram-negative group, *Pseudomonas aeruginosa* is considered a common causative agent, but other pseudomonal species are extremely rarely found as a culprit in IE.

*Pseudomonas stutzeri* is a Gram-negative bacillus that belongs to the *Pseudomonas* genus. It plays a vital role in various ecosystems, with different beneficial applications [1]. Nonetheless, *P. stutzeri* can be harmful, as it is capable of infecting the human body. It is considered an opportunistic organism and can affect different systems in the human body [2], albeit rarely. To the best of our knowledge, *P. stutzeri* was previously shown to underlie IE in only six cases in the literature from all around the globe. Herein, we present a unique case of recurrent IE caused by *P. stutzeri* 6 weeks after complete remission in a 51-year-old man with a bioprosthetic mitral valve.

## Case report

A 51-year-old arab man, known to have systemic lupus erythematosus (SLE) complicated with lupus nephritis. He underwent renal transplantation twice in 1997 and 2019 and was maintained on prednisone 5 mg once daily and tacrolimus 1 mg twice per day. He also is known to have severe mitral valve stenosis secondary to rheumatic heart disease. His family history was unremarkable; particularly, there was no history of SLE or rheumatic heart diseases. He works as a businessman, is married, and has two children. He lives in a private house and does not consume alcohol or tobacco.

At the age of 45 years, a successful mechanical mitral valve replacement (MVR) procedure was performed utilizing Medtronic valve size 29 mm. He was maintained on warfarin 4 mg daily with a subtherapeutic internal normalization ratio (INR) due to noncompliance issues. Four years later, he was found to have a stuck mechanical mitral valve leaflet by a routine transthoracic echocardiography, which was confirmed by transesophageal echocardiography (TEE) and fluoroscopy. He was asymptomatic clinically, and his physical examination was remarkable for an audible click of the first heart sound (S1), normal second heart sound (S2), and a grade 2/6 pan-systolic murmur over his apical area. As a result, a redo MVR was performed utilizing a bioprosthetic valve (Epic St. Jude bioprosthesis size 31 mm).

One month later, he presented to emergency room complaining of subjective fever, reaching up to 39 °C, associated with chills for a 2-day duration. His heart rate was 86 beats per minute and blood pressure was 118/67 mmHg. His physical examination revealed a normal pulse and no splinter hemorrhages, Osler nodes, or Janeway lesions. His precordial examination revealed normal and audible S1 and S2 with grade 3/6 pansystolic murmur over his apical area that radiated to his axilla. His higher mental function and neurological examination were normal, and his ophthalmological examination excluded the presence of Roth’s spots. His dental examination revealed few dental caries, and he denied any recent dental or gingival procedures or manipulations. Blood cultures were obtained for investigations, and the patient was discharged on oral paracetamol 650 mg orally every 4 hours as needed. His blood cultures came back with a positive result of isolated *P. stutzeri*.

The patient was called back and admitted for urgent investigations, where he underwent a transthoracic echocardiography that revealed vegetations on the bioprosthetic mitral valve. Subsequently, a TEE was performed and revealed two medium-sized vegetations attached to both atrial and ventricular sides of the mitral prosthesis. They measured approximately 5 × 4 mm and 7 × 6 mm, respectively, morphologically consistent with prosthetic valve endocarditis (PVE), rather than Libman–Sacks Endocarditis. Rheumatological investigations were negative, and he had stable and inactive SLE. The patient was treated as a case of PVE with intravenous piperacillin/tazobactam 4.5 g every 6 hours for 3 weeks as an inpatient and was discharged thereafter in stable condition on oral ciprofloxacin 400 mg twice a day to complete a total duration of 6 weeks, based on his blood culture sensitivity results (Table 1).

**Table 1:** Susceptibility profile of the *P. stutzeri* isolate

Tested antibiotics	Reported susceptibility
Ceftazidime	Sensitive
Cefepime	Sensitive
Gentamicin	Sensitive
Tobramycin	Sensitive
Imipenem	Sensitive
Meropenem	Sensitive
Ciprofloxacin	Sensitive

Two months later, he again presented to emergency room complaining of fever, reaching 38.3 °C, and chills. His symptoms were associated with dysarthria and aphasia. His physical examination was notable for a heart rate of 87 beats per minute and blood pressure of 136/78 mmHg. A reduced power of grade 2 over 5 was noted in his right upper and right lower limbs. His cardiovascular examination was remarkable for a normal and audible S1 and S2 with a grade 4 over 6 pansystolic murmur over his apical area. There were no peripheral stigmata of infective endocarditis. While in emergency room, he lost consciousness and was intubated and placed on mechanical ventilation. Computed tomography (CT) scan of his brain revealed a completely occluded left middle cerebral artery (MCA); unfortunately, thrombolysis was not feasible because of a high INR level (4.9). Subsequent brain magnetic resonance imaging (MRI) revealed multiple brain and brainstem lesions, which were suggestive of underlying showering emboli.

**Fig. 1:**
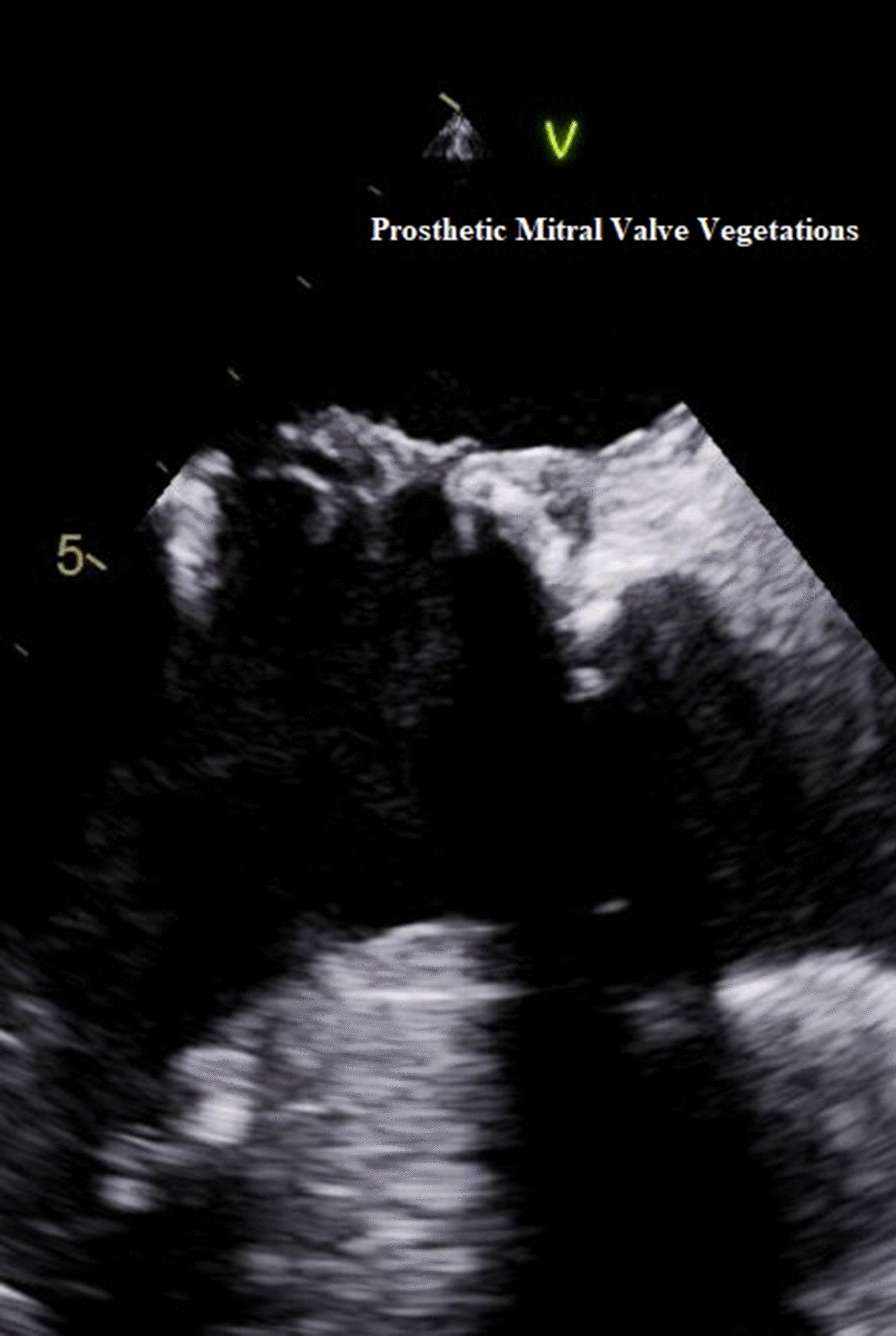
Transesophageal echocardiography showing the prosthetic mitral valve with vegetations attached to the atrial surface

His routine blood work-up was unremarkable, apart from leukocytosis of a 16.3 × 10^9^ per liter and a positive blood culture growing the same organism, *P. stutzeri*. His hemoglobin level, platelet count, and renal and hepatic profiles were all within normal range. His serological markers were negative. Intravenous ceftazidime 2 g every 12 hours was started, and the patient underwent TEE, which revealed several mobile masses attached to the atrial side and coating the bioprosthetic mitral valve consistent with vegetations (Fig. 1). The vegetations were extending to involve the aortomitral fibrosa and the aortic valve causing severe degree of aortic regurgitation and suggesting an ongoing abscess formation.

**Fig. 2:**
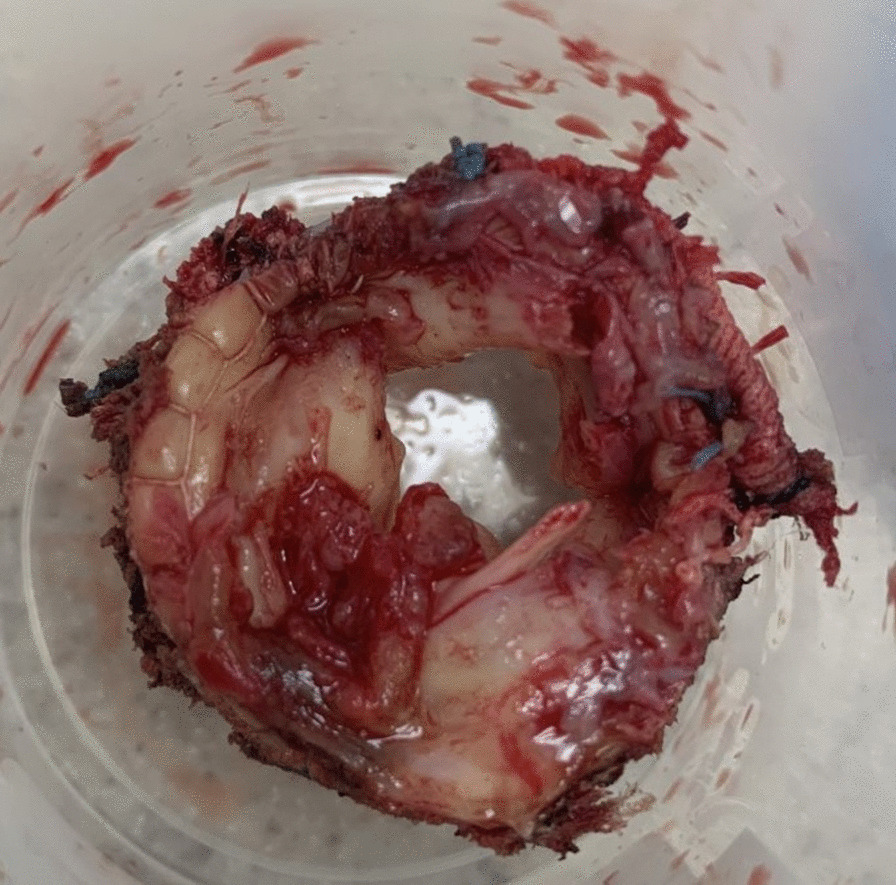
The excised mitral valve prosthesis showing leaflets thickening with multiple gross masses consistent with vegetations

The case was discussed in a combined cardiology/cardiac surgery meeting, and the decision was made for the patient to undergo a second redo MVR. After redo sternotomy, dissection of adhesions, and cardiopulmonary bypass connection, the prosthetic valve was explored. The prosthetic mitral valve leaflets were thickened and coated with multiple gross masses consistent with vegetations (Fig. 2); similar masses were also noted on the aortic valve cusps. The prosthetic mitral valve was excised and replaced with a 33 mm bioprosthesis (Perimount Magna Ease); the aortic valve was also excised and replaced with a size 25 mm bioprosthesis (Perimount Magna Ease). The excised valves and masses were sent for histopathological examination, which confirmed the presence of vegetations, and the surgical cultures grew the same organism. The postoperative period was unremarkable, and the antimicrobials regimen were continued throughout his hospital stay.

The patient was seen at a follow-up visit 6 months after his surgery, where his sternal wound was intact and revealed no signs of active infection or inflammation. He had an intact higher mental function with no aphasia or dysarthria, and his right upper and lower limbs power was 4 over 5. His physical examination was otherwise unremarkable.

## Discussion

This is a case of a 51-year-old gentleman, who has SLE complicated by lupus nephritis and who underwent renal transplantation twice. He underwent mitral valve replacement and 4 years later was diagnosed with prosthetic valve endocarditis caused by *P. stutzeri* complicated with a left MCA stroke. He underwent a redo MVR successfully and made a reasonable recovery on 6-month follow-up.

Prosthetic valve infective endocarditis following surgical valve replacement occurs in 1–3% of patients in the first year after surgery and is associated with high morbidity and mortality [3]. Gram-negative pathogens account for less than 10% of all cases of infective endocarditis worldwide [4, 5]. While *Pseudomonas* species rarely cause prosthetic valve endocarditis, *Pseudomonas aeruginosa* is the most frequently implicated pathogen in this group [4]. *P. stutzeri* with its low virulence and high susceptibility to antibiotics has not been frequently implicated in prosthetic valve endocarditis [6].

*Pseudomonas stutzeri* was first described by Burri and Stutzer in 1895 [7]. This Gram-negative, rod-shaped, aerobic, and oxidative-positive bacterium is naturally found in soil and water [2]. True human infections with this bacterium occur particularly in immunocompromised patients with chronic comorbidities, a history of previous surgery, previous trauma, or skin infections, and are frequently related to prosthetic devices. To date, many cases of *P. stutzeri* infection have been reported in the literature, including bacteremia, pneumonia, osteomyelitis, arthritis, and ocular infections [6, 8]. It has also been hypothesized that *P. stutzeri* infections may develop more easily in inflamed tissue, as was observed in two patients being treated for pulmonary tuberculosis with considerable parenchymal destruction who developed complicating superinfections with *P. stutzeri* [9, 10].

*Pseudomonas stutzeri* is rarely reported as a pathogenic agent of infective endocarditis, with only six cases having been reported previously in the literature (Table 2). Cases were reported from Israel, Spain, France, Lebanon, and Saudi Arabia. Four cases of *P. stutzeri* prosthetic valve infective endocarditis occurred, on average, 4 years post-cardiac surgery, while two cases affected a native valve in patients with no prior surgical history. The source of infection could not be identified in any of the cases. All patients were successfully treated with surgery and antibiotics and survived, except one patient [8, 11–14].

**Table 2:** Previous cases of *P. stutzeri* infective endocarditis

Cases	Year of publication	Country	Valve	Antibiotics	Surgery	Outcome	Time after cardiac surgery
Rosenberg *et al.* [15]	1987	Israel	Prosthetic mitral	Tobramycin and mezlocillin	Not done	Cured	2 years
López *et al.* [11]	2002	Spain	Native aortic	Aztreonam, cefotaxime, and ceftriaxone	Aortic valve replacement	Cured	Not applicable
Grimaldi *et al.* [12]	2008	France	Prosthetic aortic	Cefotaxime, ciprofloxacin, and doxycycline	Not done	Cured	6 and 10 years
Shalabi *et al.* [8]	2017	Lebanon	Prosthetic aortic and mitral	Ceftazidime	Aortic valve replacement	Cured	3 years
Halabi *et al.* [13]	2018	Lebanon	Prosthetic aortic	Ceftazidime	Aortic and tricuspid valves replacement	Deceased	26 days
Alwazzeh *et al.* [14]	2020	Saudi Arabia	Native mitral and aortic	Vancomycin, ceftriaxone, and cefepime	Mitral and aortic valves replacement	Cured	Not applicable

A striking geographical distribution of *P. stutzeri* infections has been observed, with 62% of all globally reported cases being detected in the Mediterranean Basin, most frequently in Israel (*n* = 30), Spain (*n *= 5), Italy (*n* = 3), and Turkey (*n* = 3). It remains unclear whether these observations relate to higher rates of contaminated medical equipment and devices in this region or whether there are specific biological factors favoring the growth of *P. stutzeri* in such environments. Further investigations are warranted to elucidate these associations [8].

There are no data comparing monotherapy with combination therapy for *Pseudomonas aeruginosa* endocarditis, mainly due to the rarity of the condition. Recommendation to use combination therapy with two antipseudomonal antibiotics including an aminoglycoside is based mainly on expert opinion and observational studies. There are no data regarding the optimal treatment of *P. stutzeri* endocarditis [13].

In our case, the source of infection and the portal of entry could not be identified. The patient underwent a redo mitral valve replacement, and a month later he presented with symptoms suggestive of a prosthetic valve endocarditis. The diagnosis was confirmed by a positive blood culture that grew *P. stutzeri* and a transesophageal echocardiography. The isolated organism was susceptible to a wide range of antibiotics as in previously reported cases, so the decision was made to treat him with a 6-week course of antibiotics. Unfortunately, and despite completing the full antibiotics course, he relapsed 2 months later, requiring a more aggressive management with combined antibiotics and a second redo mitral valve replacement. Unlike the majority of the previous reported cases of *P. stutzeri* infective endocarditis, this is a reported case from a country that does not belong to Mediterranean Basin. An indolent clinical course was also well documented in the Grimaldi *et al.* case with relapse after 4 years [12]. To the best of our knowledge, this is the second case of fulminant early prosthetic valve endocarditis occurring only 1 month post-cardiac surgery with relapse despite a complete antibiotics course.

## Conclusion

*Pseudomonas stutzeri*, even though frequently considered as contaminant, can give rise to potentially severe infections, including endocarditis. Those infections are being identified and recognized more frequently. The source of infection remains unclear. *P. stutzeri* isolates remain susceptible to a wide array of antibiotics. Endocarditis typically occurs years after cardiac surgery with a good overall outcome. Our case proves that early infection and relapse despite the optimum antibiotics course are possible outcomes of *P. stutzeri* endocarditis.

## Data Availability

Not applicable.
